# Supplementary material for: Hypothalamic Median Eminence Thyrotropin-Releasing Hormone-Degrading Ectoenzyme Activity Is Dispensable for Basal Thyroid Axis Activity in Lean Rodents
Source: Cells. 2025 May 15;14(10):725. doi: 10.3390/cells14100725 (PMC12110239; doi:10.3390/cells14100725)
Supplement: Supplementary file 1 [file cells-14-00725-s001.zip › cells-3503163-supplementary.pdf]

SUPPLEMENTARY MATERIALS FOR:

Hypothalamic Median Eminence Thyrotropin-Releasing Hormone-Degrading Ectoenzyme Activity Is Dispensable for Basal Thyroid Axis Activity in Lean Rodents

Adair Rodríguez-Rodríguez, Rosa María Uribe, Antonieta Cote-Vélez, Patricia Joseph-Bravo and Jean-Louis Charli *

Departamento de Genética del Desarrollo y Fisiología Molecular, Instituto de Biotecnología, Universidad Nacional Autónoma de México (UNAM), Avenida Universidad 2001, Cuernavaca 62210, Mexico

LIST OF SUPPLEMENTARY MATERIALS

**Supplementary Table S1**	Pages 3, 4
**Supplementary Table S2**	Page 5
**Supplementary Table S3**	Page 6
**Supplementary Table S4**	Page 7
**Supplementary Table S5**	Page 8
**Supplementary Table S6**	Page 9-10
**Supplementary Figure Legends**	Pages 11-13
**Supplementary Figure S1**	Page 14
**Supplementary Figure S2**	Page 15
**Supplementary Figure S3**	Page 16
**Supplementary Figure S4**	Page 17
**Supplementary Figure S5**	Page 18
**Supplementary Figure S6**	Page 19

Supplementary Table S1. List of experiments performed.

Experiment	Objective	Number of animals per group	Time (weeks) between AAV injection and sacrifice
**Mice**			
**Vimentin immunohistochemistry**	Observation of vimentin in ME of WT and *Trhde* KO mice	4	
**β-gal histochemistry**	Observation of β-gal activity in WT and *Trhde* KO mice	2	
**β-gal immunofluorescence**	Observation of β-gal immunofluorescence in WT and *Trhde* KO mice	2	
**Cohorts 1 to 8**	Molecular, behavioral and endocrine phenotyping	1-10	
**Rats**			
**Immunohistochemistry post PFA/ acrolein perfusion**	Observation of TRH-DE and its relationship with tanycytes and TRH terminals in the ME	3	
**3V injection of 8 different AAV serotypes. Analysis of distribution of transduced cells in brain**	Identification of an AAV serotype suitable for transducing median eminence tanycytes	3	2 (3 and 4 not shown)
**1**	Effect of 3V injection of AAV1-TRH-DE or -TRH-DEt on PVH *Trh* expression, pituitary expression and TRH-DE immunohistochemistry	5-6; 2-3 for immunohistochemistry	
**2**	Effect of 3V injection of AAV1-TRH-DE on serum TSH concentration at baseline and after cold exposure	3-6	2
**3**	Effect of 3V injection of AAV1-TRH-DE on HPT axis activity	2-4	2, 3 and 4

**4**	Effect of 3V injection of AAV1-TRH-DEt on HPT axis activity	5	2
**5**	Effect of 3V injection of two increasing loads of AAV1-TRH-DE on HPT axis activity	5	2
**6**	Effect of 3V injection of AAV1-TRH-DE or TRH-DEt on serum leptin and triglyceride concentrations	7-8	2
**7**	Effect of injection in SON of AAV1-TRH-DE on HPT axis activity	5	2

Supplementary Table S2. List of primary and secondary antibodies used for immunofluorescence.

Antibody	Final Dilution	Source	Catalog No.
**Goat anti-mouse IgG Alexa 647**	1:500	Jackson Immuno Research	115-605-062
**Goat anti-rabbit IgG Alexa 488**	1:500	Jackson Immuno Research	111-545-003
**Goat anti-chicken IgY Alexa 647**	1:500	Jackson Immuno Research	103-625-155
**Bovine anti-goat IgG Cy3**	1:500	Jackson Immuno Research	805-165-180
**Rabbit anti-human TRH-DE[2]**	1:500	Abexxa	abx322700
**Chicken anti-vimentin**	1:4000	Merck	AB5733
**Rabbit anti-bacterial β-gal**	1:5000	Invitrogen	A11132
**Sheep anti-TRH[1]**	1:5000	[83]	

[1] generated using a TRH-acrolein-bovine serum albumin conjugate, a gift from Dr. Csaba Fekete laboratory. [2] generated by immunizing with a recombinant TRH-DE fraction of the protein (aa 62-200).

Supplementary Table S3. List of primers used for PCR, RT-PCR and RT-qPCR protocols.

Species	Gene	Used for	Forward 5´->3´	Backward 5´->3´
**Rat**	**Tshb**	RT-PCR	TCTGCGCTGGGTATTGTATG	CGTTCTGTAGGTGAAGTCTCTG
	**Trhr**		GCACATGAGAACCGCTACAA	CCATAGACCCAGGAACCATAGA
	**Prl**		CACTTCTTCCCTAGCTACTCCT	AGTTGAAACAGAGGGTCATTCC
	**Hprt**		GGCCAGACTTTGTTGGATTTG	CTTTCGCTGATGACACAAACAT
	**Thrb**		GACTGGAAGCTGGTAGGAATG	GGATGAGGTGTGAGGATGTTT
	**Trh**		AGAGGGAGAGGGTGTCTTAAT	GCTAGTGAAGGGAACAGGATAG
	**Dio2**		GGAGGCAAGTCAGTCATTGT	CCACAGGAGCAGAGATCTAAAG
	**Rplp0**		GAGCGATGTGCAGCTGATAAA	ATGATCAGCCCGAAGGAGAA
**Mouse**	**Trhde^{tm1Lex}**	PCR	GCAGCGCATCGCCTTCTATC	CCACTGTAACCTGAGAAGTTG
	**Trhde**		CTCTCGGACCCGTGGGCTG	GCATCAAATTGTAGTGCAGCG
	**Trh**	RT-qPCR	GATTCTGGAGCCTTGCAGAC	GGGGATACCAGTTAGCACGA
	**Trhde**		TCAATCAAACCGGCTACTT	CCCTCCGTAGCTCCTCATGT
	**Dio1**		ACGTGCCTCAGCTCTAGGGG	AGCAGAACATGCCTGCCTCTTG
	**Actb**		TCATGAAGTGTGACGTTGACATCCGT	CCTAGAAGCATTTGCGGTGCACGATG
	**Hprt**		GGCCATCACATTGTGGCCCT	AAGTCTGGGGACGCAGCAACT
	**Ppia**		CGCGTCTCCTTCGAGCTGTTT	AGATGGGGTAGGGACGCTCTC

Supplementary Table S4. Additional effects of *Trhde* genotype on C57BL/6NJ mice phenotype.

	**Male**			**Female**		
	**WT**	**HT**	**KO**	**WT**	**HT**	**KO**
**Serum TRH concentration[1] after 1 ng/g TRH**	27.7 ± 2.4 (7)	43.8 ± 4.9 (10)	94.5 ± 10.3 (7) **** &&&&	32.2 ± 7.5 (6)	50.4 ± 4.4 (17)	95.6 ± 18.1 (5) **** &&&&
**Serum TRH concentration[1] after 10 ng/g TRH**	173 ± 46.2 (10)	415 ± 125 (12)	993 ± 128 (9) **** &&&&	256 ± 53.7 (10)	558 ± 74 (10)	1480 ± 185 (10) **** &&&&
**Brain APN activity[2]**	3730 ± 192 (4)	4150 ± 439 (4)	4530 ± 252 (4)	ND	ND	ND

Data (mean ± SEM (n)) correspond to one cohort. [1] pg TRH 15 min after ip injection. [2] pMoles βNA/min/mg prot. ND: not determined. Statistical tests: One way ANOVA. ****: p<0.0001 compared to HT; &&&&: p<0.0001 compared to WT.

Supplementary Table S5. Patterns of transduction found after delivery of different AAV serotypes into the third ventricle of male rats.

Serotype	Positive cell type in ME	Level of ME expression	Hypothalamic nuclei positive neurons	Choroid plexus	Meningeal cells	Subcommisural organ	Anterior pituitary	Posterior pituitary
**scAAV1[a]**	tan, um	+++	SON	-	++	-	-	++
**scAAV2[a]**	tan, var	+	SON, peVN	-	++	-	-	+
**AAV4[b]**	var	+++	-	++	++	+++	-	-
**scAAV5[a]**	-	+	-	+++	-	-	-	-
**scAAV6[a]**	-	-	SON	+++	++	+++	-	+++
**scAAV8[a]**	tan, var, um	+	SON, peVN	-	++	-	-	+++
**scAAV9[a]**	tan, var, um	+	SON, DMH, pevN	-	++	-	-	+++
**scAAVD/J[a]**	-	-	SON, peVN	-	++	-	-	+++

[a]: 4.5×10^{9} viral genomes injected; [b]: 8.75×10^{10} viral genomes injected; tan: cells with tanycyte morphology; um: cells with undetermined morphology; var: varicosities; peVN: periventricular.

Supplementary Table S6. Results of statistical analyses.

Experiment	Variable	Figure	Main test	Source of variation	F or t value	P value	Post hoc test	Groups	Post hoc P
**Mice cohorts 1 to 8**	Brain *Trhde* expression	2A	Two-way ANOVA	Interaction	F(2,54) = 1.004	0.3732	Tukey	Male WT vs. HT	< 0.0001
				Sex	F(1,54) = 4.0360	0.0496		WT vs. KO	< 0.0001
				Genotype	F(2,54) = 126.2	< 0.0001		Female WT vs. HT	0.0025
								HT vs. KO	< 0.0001
								WT vs. KO	< 0.0001
	Pituitary *Trhde* expression	2B	Two-way ANOVA	Interaction	F(2,25) = 0.002	0.9973	Tukey	Male WT vs. HT	0.001
				Sex	F(1,25) = 0.012	0.9108		WT vs. KO	< 0.0001
				Genotype	F(2,25) = 53.67	< 0.0001		Female WT vs. HT	0.0015
								WT vs. KO	< 0.0001
	ME TRH-DE activity	2C	Two-way ANOVA	Interaction	F(2,12) = 0.4877	0.6257	Tukey	Male WT vs. HT	0.0487
				Sex	F(1,12) = 0.1190	0.7361		WT vs. KO	0.0013
				Genotype	F(2,12) = 24.34	0.0001		Female WT vs. KO	0.0123
	Thyroliberinase activity	2D	One-way ANOVA		F(2,10) = 35.54	0.0001	Tukey	WT vs. HT	0.007
								WT vs. KO	< 0.0001
								HT vs. KO	0.0044
	Body weight	2E	Two-way ANOVA	Interaction	F(2,30) = 6.285	0.0053	Tukey	Male HT vs. KO	0.0001
				Sex	F(1,30) = 40.80	< 0.0001			
				Genotype	F(2,30) = 9.119	0.0008			
	Food intake	2F	Two-way ANOVA	Interaction	F(2,30) = 5.497	0.0092	Tukey	Male HT vs. KO	0.0007
				Sex	F(1,30) = 5.125	0.031			
				Genotype	F(2,30) = 5.728	0.0078			
	Vimentin rami-	2O	Mann Whitney non			0.002			

	fications		parametric						
**Rat Experiment 2**	TSH after cold	3B	Mann Whitney non parametric			0.0357			
**3**	ME TRH-DE activity	3C	Two-way ANOVA	Interaction	$F(1,10) = 4.451$	0.0611	Bonferroni	Week 2 Veh. vs. AAV1-TRH-DE	0.0463
				Week	$F(1,10) = 2.235$	0.1658			
				Treatment	$F(1,10) = 4.379$	0.0629			
	TSH levels	3D	Two-way ANOVA	Interaction	$F(2,16) = 0.5160$	0.6065	Bonferroni	Week 3 Veh. vs. AAV1-TRH-DE	0.4609
				Week	$F(2,16) = 3.510$	0.0545			
				Treatment	$F(1,16) = 13.66$	0.0020			
**4**	ME TRH-DE activity	3G	Student's t test		t=2.597, df=7	0.0359			
	TSH levels	3H	Student's t test		t=3.064, df=7	0.0182			
**5**	Relative ME TRH-DE activity	4A	Mann Whitney non parametric			0.0001			
	Prl levels	4E	Student's t test		t=2.767, df=18	0.0127			
	Trhr levels	4G	Student's t test		t=2.479, df=18	0.0233			
**7**	ME TRH-DE activity	6A	Mann Whitney non parametric			0.0159			
	Neuro TRH-DE activity	6C	Mann Whitney non parametric			0.0079			
	TSH levels	6D	Student's t test		t=1.908, df=8	0.0928			

Supplementary Figure Legends

Figure S1. Activity and immunoreactivity of TRH-DE in the rat brain and serum. [A] TRH-DE specific activity (pmoles/min x mg of protein or x 10 μL of serum) in adenohypophysis (Ad), neurohypophysis (Ne), median eminence (ME), serum (Se), and whole brain (ABr) of adult male rats. **[B]** Development of TRH-DE specific activity in the rat ME between postnatal days 10 and 60. Experiment 1. **[C]** Immunofluorescence showing distribution of TRH-DE immunoreactivity in layer V of retrospenial granular cortex (RsGC). **[D]** Immunofluorescence showing distribution of TRH-DE immunoreactivity in pyramidal neurons (py) of the ventral CA3 region of the hippocampus (vCA3). **[E]** Intense immunodetection of TRH-DE in the internal (IZ) and the external zone (EZ) of the ME in rats 3V injected with $8.75x10^{10}$ vg of AAV1-TRH-DE. **[F]** TRH-DE immunoreactive neurons (marked with a white arrow) can be traced along the trajectory of a misplaced injection of $8.75x10^{10}$ vg of AAV1-TRH-DEt in the ARC**. [G]** TRH-DE immunoreactive neurons (marked with a white arrow) can be traced along the trajectory of an injection of $8.75x10^{10}$ vg of AAV1-TRH-DEt in the cortex. **[H]** The distribution of TRH-DE immunoreactivity in the posterior part of the rat medio basal hypothalamus (green) is like that seen in the medial part shown in Fig. 1. I-VI: layers 1 to 6 of RsGC; 3V: third ventricle; gl: gliosis. Data are mean ± SEM.

Figure S2. Histochemistry of β-gal activity in the *Trhde* KO adult mouse brain. β-gal activity was detected in the cortex, hippocampus and selected regions of the medial basal hypothalamus as the arcuate nucleus (Arc), dorsomedial (DMH) and lateral hypothalamus (LH). Staining was not detected in the ME.

Figure S3. Immunohistochemistry of β-gal in the *Trhde* KO adult mouse brain. β-gal immunoreactivity was detected in the cortex, hippocampus and less prominently in the ependymal layer of the median eminence and in the arcuate nucleus. 3V: third ventricle, DG: dentate gyrus, CA3: CA3 region of hippocampus, Tan: tanycyte, pc: portal capillary.

Figure S4. Transduction sites detected with intra 3V injection of distinct AAV serotypes in rats. [A] Characterization of the injection site. Cannula (red line) used to inject stains, vehicle or AAV in the 3V. Injection of trypan blue through a cannula stained the ME (blue arrow at the base of the brain)**. N**on-specific fluorescence due to gliosis detected with a wide green fluorescence filter indicates the position of the cannula in the 3V (blue arrow). **[B]** Schematic representation of scAAV or conventional AAVs used to evaluate the transduction in ME cells of the rat. **[C]** Examples of serotypes that show extrahypothalamic transduction in the sub-commisural organ (sco) and the choroid plexus (ChP). **[D]** Serotypes that show variable degree and localization of GFP expression in the ME. scAAV1-GFP ($4.5x10^9$ vg) and to a lower degree scAAV2-GFP ($4.5x10^9$ vg) injected into the 3V transduced multiple ME cell types, including cells with tanycyte morphology and vimentin expression; AAV4-GFP ($8.75x10^{10}$ vg) injected into the 3V only transduced putative ME nerve terminal buttons. d3V: dorsal third ventricle; tb: terminal buttons. **[E]** Effect of stereotaxic surgery and delivery of AAV1-TRH-DE ($4.5x10^9$ vg) on body weight. Data are mean ± SEM. Statistical test in [E]: two-way ANOVA. *: $p<0.05$, **: $p<0.01$ for sham or AAV1-GFP vs intact.

Figure S5. Some AAV serotypes induced neurohypophyseal transduction when delivered into the rat 3V. **[A]** Injection of serotypes 1, 2, 6, 8 and 9 AAV-GFP ($4.5x10^9$ vg) induced expression of GFP in the posterior pituitary (NH), in compartments resembling Herring bodies (hb) and axons (ax). **[B]** These serotypes expressed GFP in the supraoptic nucleus (SON). OPT: optic tract. AH: anterior pituitary. **[C]** Experiment 3. AAV1-TRH-DE ($8.75x10^{10}$ vg) delivery in the ME did not change TRH-DE activity in the anterior pituitary. **[D]** AAV1-TRH-DE ($8.75x10^{10}$ vg) delivery in the ME transiently enhanced TRH-DE activity in the posterior pituitary. Data are mean ± SEM. Statistical test in [D]: two-way ANOVA. *: $p<0.05$ for vehicle vs AAV1-TRH-DE.

Figure S6. Effect of AAV vectors expressing TRH-DE or TRH-DEt on TRH-DE activity in COS-7 cells and in the ME. **[A]** $3x10^5$ COS-7 cells were incubated with AAV1-TRH-DE alone (1:0) at a multiplicity of infection (moi) of $1x10^4$ in 35 mm dishes or coincubated with increasing concentrations of AAV1-TRH-DEt (1:1, 1:5, 1:10 ratios). **[B]** Experiment 1. Measurement of TRH-DE

immunoreactivity showed that both injections of $8.75x10^{10}$ vg of AAV1-TRH-DE (orange) or AAV1-TRH-DEt (clear orange) enhanced detection with anti-TRH-DE antibodies along the rostrocaudal extent of the ME when compared with levels after AAV1-GFP injection (green). **[C]** 3V injection of AAV1-TRH-DE or AAV1-TRH-DEt enhanced immunodetection with anti-TRH-DE antibodies (red) in the external zone of the ME. Experiment 3. Stereotaxic surgery and delivery of $8.75x10^{10}$ vg of AAV1-TRH-DE did not affect **[D]** the rate of body weight increase and **[E]** thyroliberinase activity, when compared to rats injected with vehicle. Experiment 4. Stereotaxic surgery and delivery of $8.75x10^{10}$ vg of AAV1-TRH-DEt did not affect **[F]** the rate of body weight increase and **[G]** thyroliberinase activity, when compared to rats injected with AAV1-GFP. Data are mean ± SEM.

Figure S7. Additional aspects of rat physiology were not affected by AAV1-TRH-DE or AAV1-TRH-DEt delivery in the 3V of rats in standard conditions. Additional correlations between variables. Experiment 5. **[A]** AAV1-TRH-DE injection ($8.75x10^{10}$ vg) did not change serum TSH concentration compared to AAV1-GFP ($8.75x10^{10}$ vg) injected rats. Two weeks after delivery of $8.75x10^{10}$ vg of AAV1-TRH-DE or AAV1-TRH-DEt in the ME, **[B]** expression of *Trh* in the PVH and **[C]** serum triglyceride concentration were not affected. Output of experiment 6 was analyzed to find correlations between fat weight and serum leptin concentration 2 weeks after viral ($8.75x10^{10}$ vg) delivery. Strong correlations between serum leptin concentration and **[D]** retroperitoneal, **[E]** escapular or **[F]** epidydymal fat content were detected, while no effect of AAV1 viral constructions was appreciable. TRH-DE activity in the ME in the AAV1-GFP group was positively correlated with **[G]** *Prl* expression in the pituitary and **[H]** serum prolactin concentration. **[I-K]** *Dio2*, *Thrb* and *Prl* expression in the pituitary were negatively correlated with serum tT4 concentration. Only Spearman rho values and Pearson r values of significant correlations are shown. Experiment 2. **[L]** Effect of injection of vehicle or AAV1-GFP on basal serum TSH concentration. Effects of vehicle or AAV1-GFP ($8.75x10^{10}$ vg) injections over serum **[M]** TSH, **[N]** tT3 and **[O]** tT4 concentrations. Data are mean ± SEM.

Figure S1

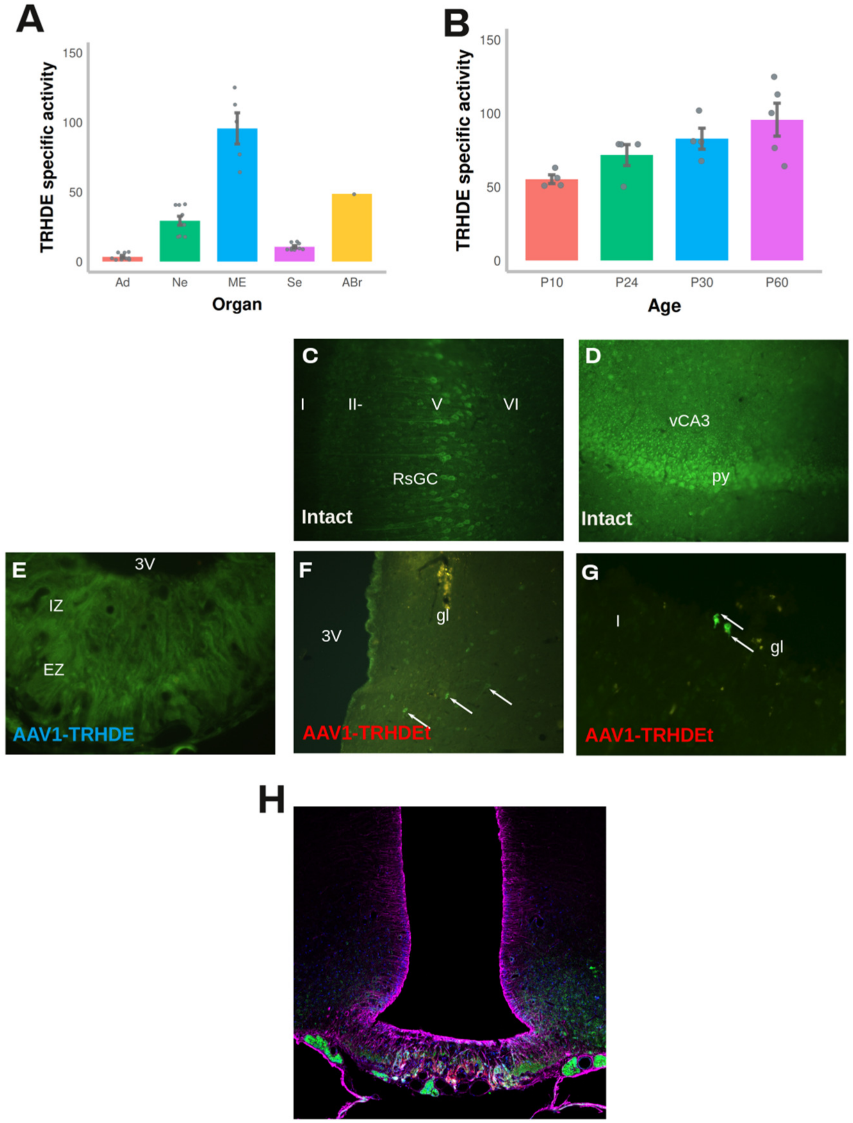
A
TRHDE specific activity
150
100
50
0
Ad
Ne
ME
Se
ABr
Organ
B
TRHDE specific activity
150
100
50
0
P10
P24
P30
P60
Age
C
I
II-
V
VI
RsGC
Intact
D
vCA3
py
Intact
E
3V
IZ
EZ
AAV1-TRHDE
F
gl
3V
AAV1-TRHDEt
G
I
gl
AAV1-TRHDEt
H

Figure S2

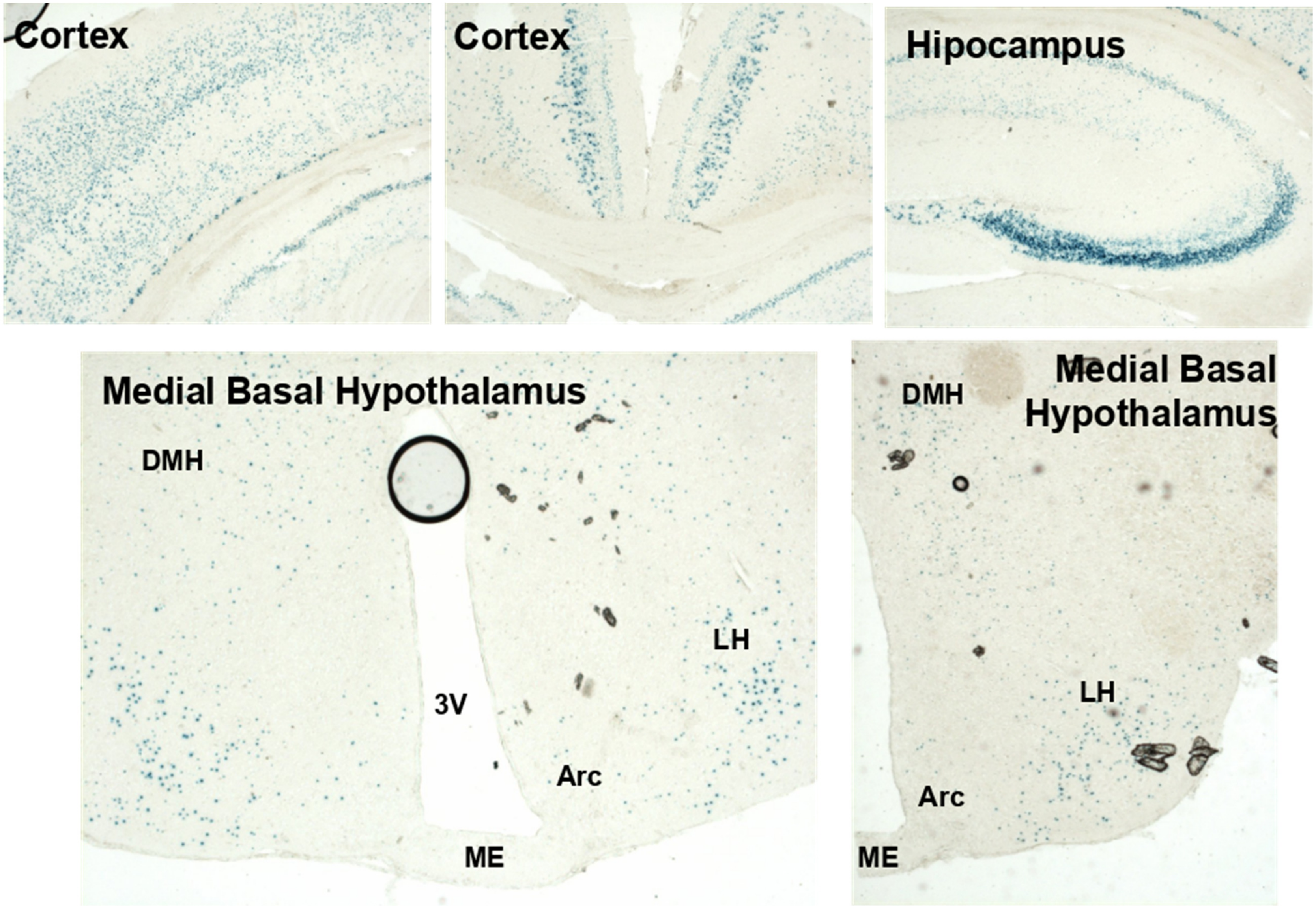
Cortex
Cortex
Hipocampus
Medial Basal Hypothalamus
DMH
3V
Arc
LH
ME
Medial Basal Hypothalamus
DMH
LH
Arc
ME

Figure S3

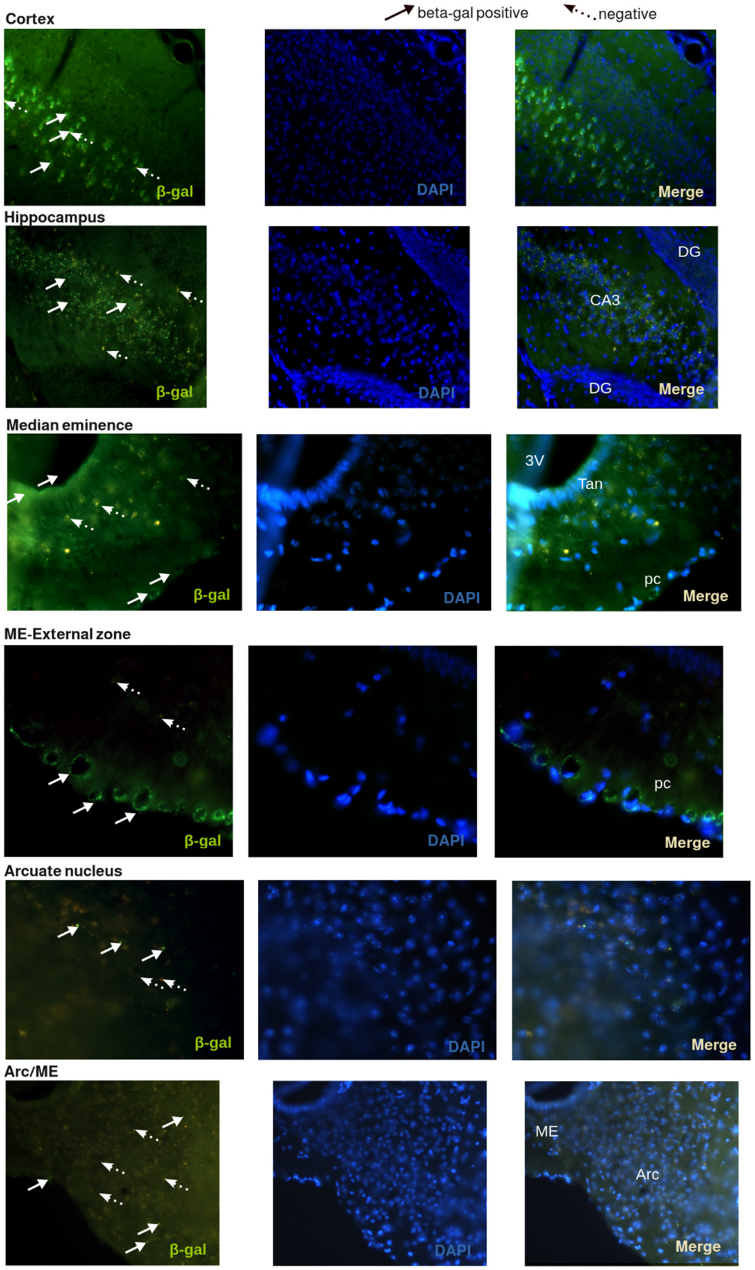
beta-gal positive
negative
Cortex
β-gal
DAPI
Merge
Hippocampus
β-gal
DAPI
DG
CA3
DG
Merge
Median eminence
β-gal
DAPI
3V
Tan
pc
Merge
ME-External zone
β-gal
DAPI
pc
Merge
Arcuate nucleus
β-gal
DAPI
Merge
Arc/ME
β-gal
DAPI
ME
Arc
Merge

Figure S4

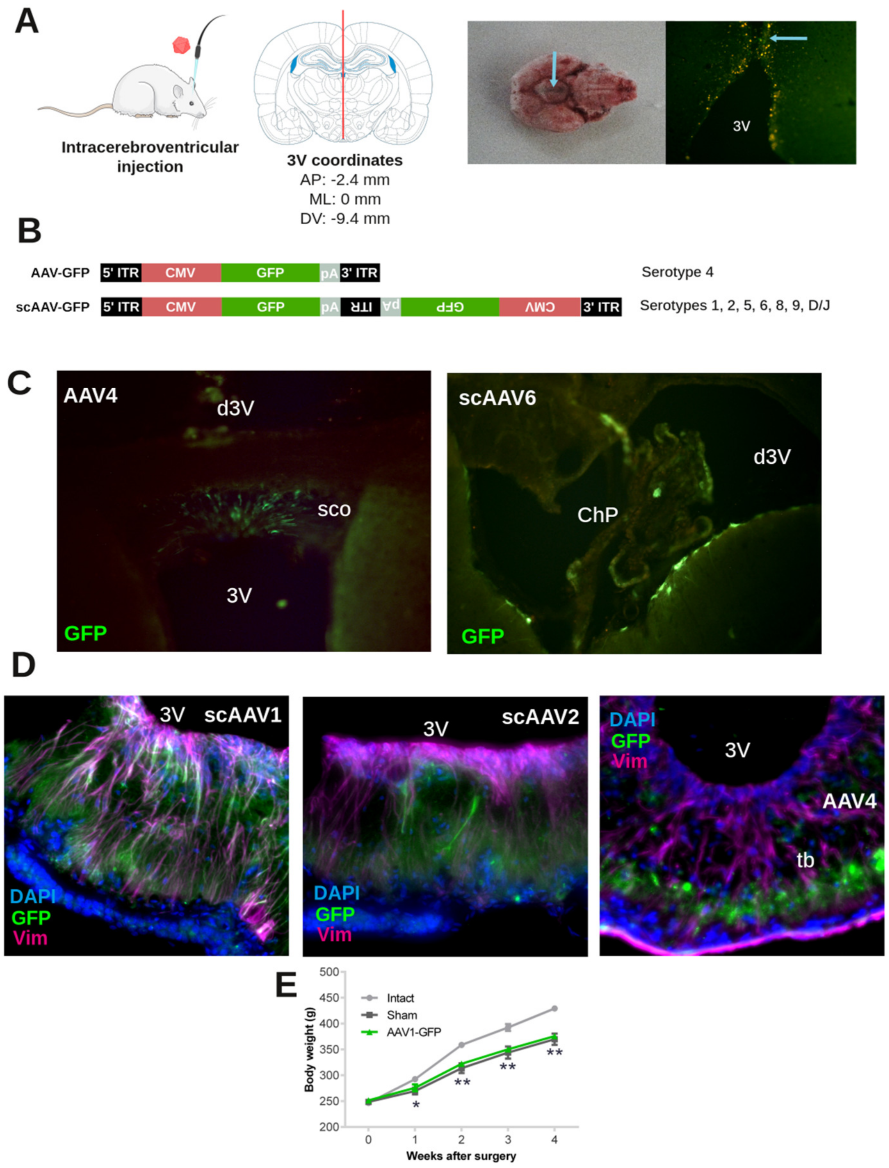
A
Intracerebroventricular injection
3V coordinates
AP: -2.4 mm
ML: 0 mm
DV: -9.4 mm
3V
B
AAV-GFP
5' ITR
CMV
GFP
pA
3' ITR
Serotype 4
scAAV-GFP
Serotypes 1, 2, 5, 6, 8, 9, D/J
C
AAV4
d3V
sco
3V
GFP
scAAV6
d3V
ChP
GFP
D
3V
scAAV1
DAPI
GFP
Vim
scAAV2
AAV4
tb
E
Intact
Sham
AAV1-GFP
Body weight (g)
Weeks after surgery

Figure S5

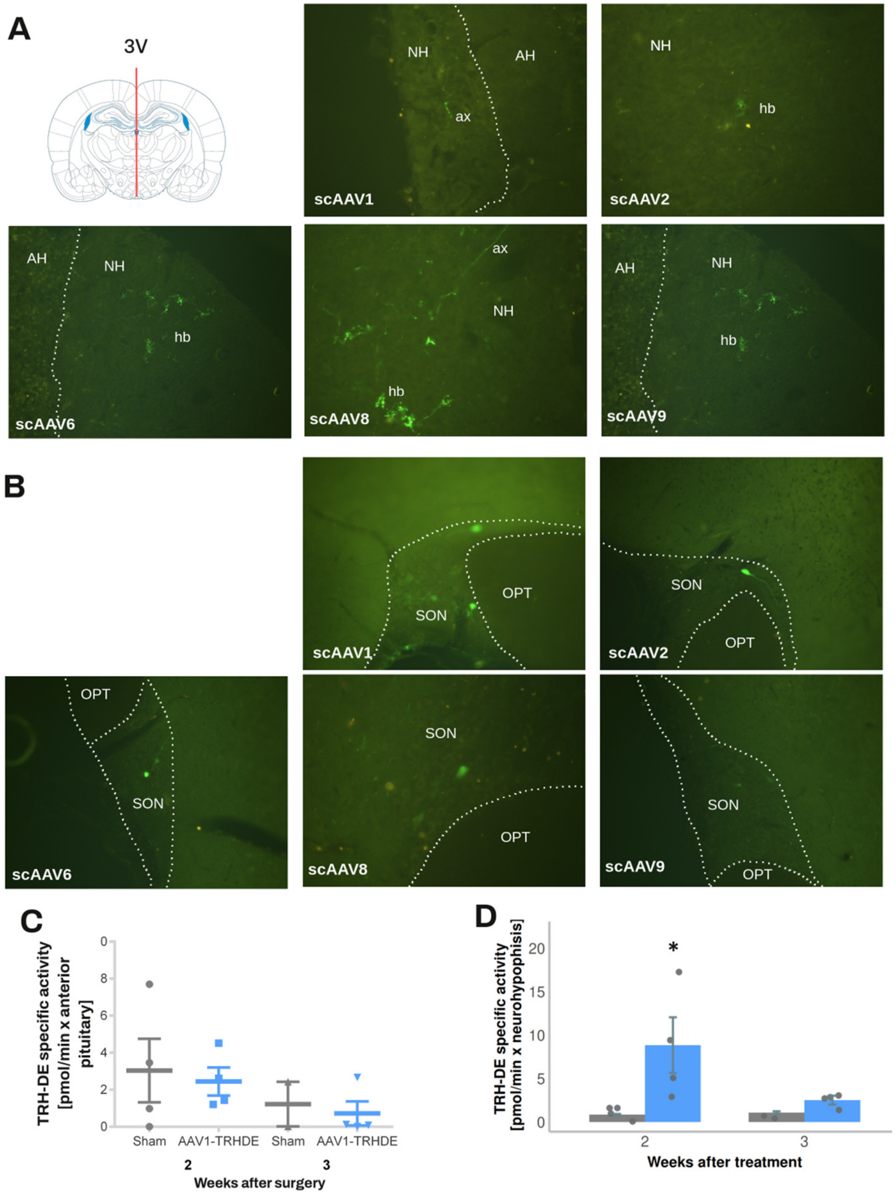
A
3V
NH
AH
ax
scAAV1
NH
hb
scAAV2
AH
NH
hb
scAAV6
ax
NH
hb
scAAV8
AH
NH
hb
scAAV9
B
OPT
SON
scAAV1
SON
OPT
scAAV2
OPT
SON
scAAV6
SON
OPT
scAAV8
SON
OPT
scAAV9
C
TRH-DE specific activity [pmol/min x anterior pituitary]
Sham
AAV1-TRHDE
Sham
AAV1-TRHDE
2
3
Weeks after surgery
D
TRH-DE specific activity [pmol/min x neurohypophysis]
*
2
3
Weeks after treatment

Figure S6

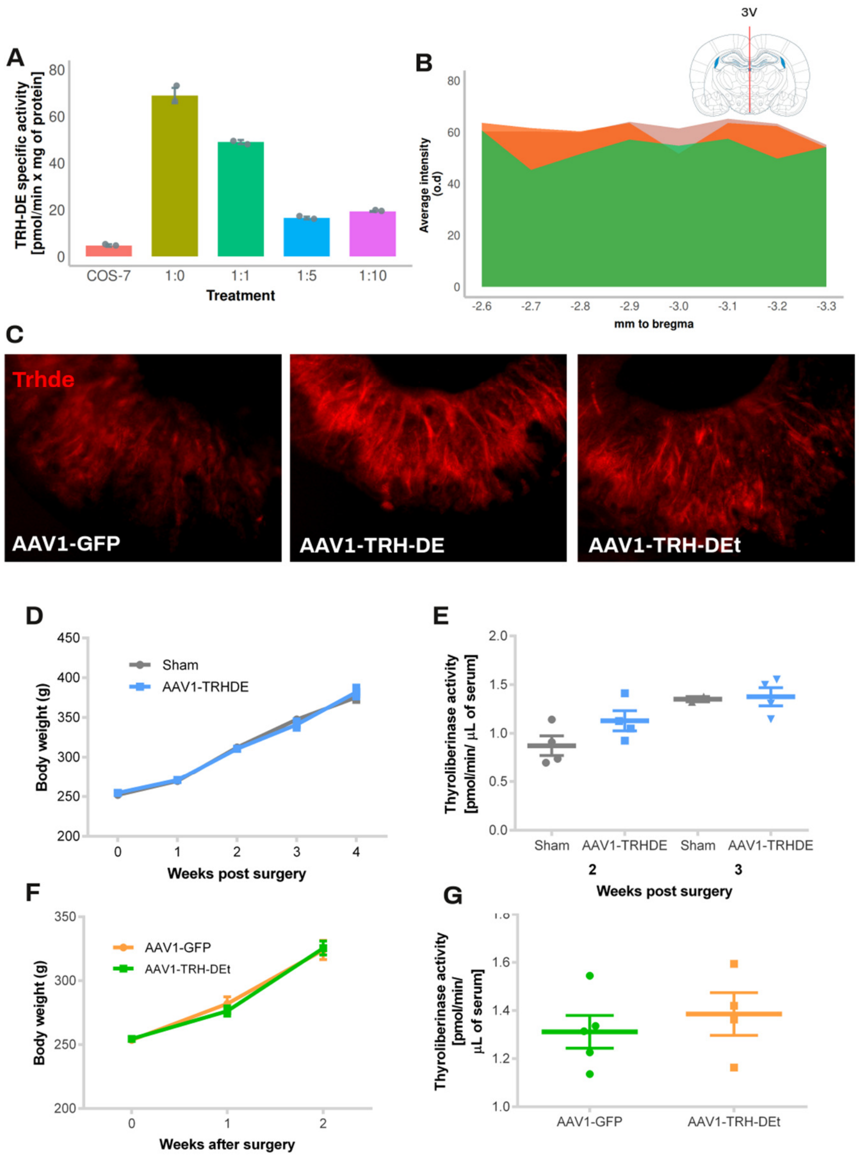

A
TRH-DE specific activity [pmol/min x mg of protein]
COS-7
1:0
1:1
1:5
1:10
Treatment
B
3V
Average intensity (o.d)
mm to bregma
C
Trhde
AAV1-GFP
AAV1-TRH-DE
AAV1-TRH-DEt
D
Sham
AAV1-TRHDE
Body weight (g)
Weeks post surgery
E
Thyroliberinase activity [pmol/min/ µL of serum]
Sham
AAV1-TRHDE
Weeks post surgery
F
AAV1-GFP
AAV1-TRH-DEt
Body weight (g)
Weeks after surgery
G
Thyroliberinase activity [pmol/min/ µL of serum]
AAV1-GFP
AAV1-TRH-DEt

Figure S7

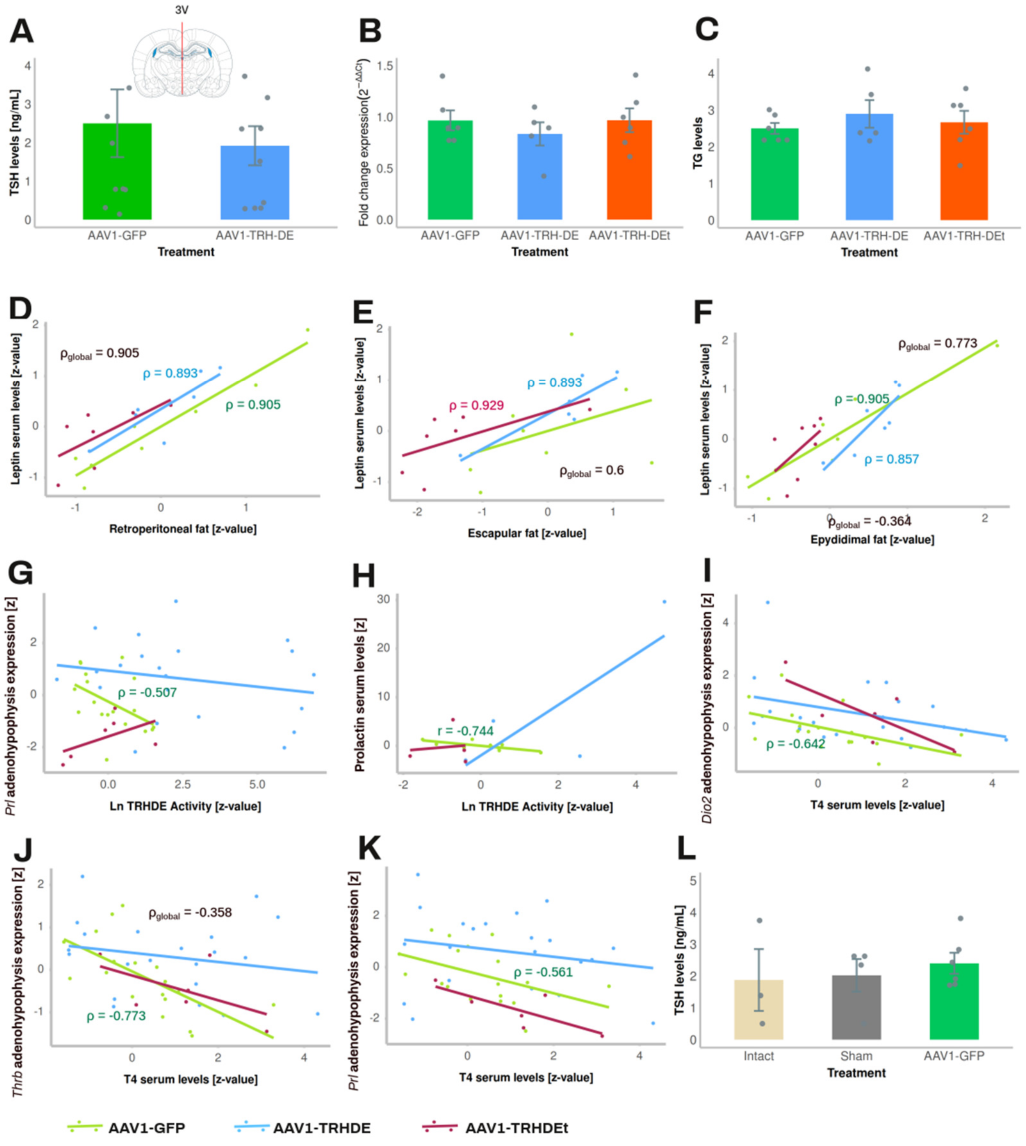
A
B
C
D
E
F
G
H
I
J
K
L
3V
TSH levels [ng/mL]
Fold change expression(2^-ΔΔCt)
TG levels
AAV1-GFP
AAV1-TRH-DE
AAV1-TRH-DEt
Treatment
Leptin serum levels [z-value]
Retroperitoneal fat [z-value]
Escapular fat [z-value]
Epydidimal fat [z-value]
ρglobal = 0.905
ρ = 0.893
ρ = 0.905
ρ = 0.929
ρglobal = 0.6
ρglobal = 0.773
ρ = 0.857
ρglobal = -0.364
Prl adenohypophysis expression [z]
Prolactin serum levels [z]
Dio2 adenohypophysis expression [z]
Ln TRHDE Activity [z-value]
T4 serum levels [z-value]
ρ = -0.507
r = -0.744
ρ = -0.642
Thrb adenohypophysis expression [z]
ρglobal = -0.358
ρ = -0.773
ρ = -0.561
Intact
Sham
AAV1-TRHDE
AAV1-TRHDEt
